# Supplementary material for: Are carbon emissions trading and green financial instruments synergistic? -Comprehensive quantitative research based on content analysis
Source: PLoS One. 2024 Mar 7;19(3):e0298601. doi: 10.1371/journal.pone.0298601 (PMC10919601; doi:10.1371/journal.pone.0298601)
Supplement: S1 Data — (ZIP) [file pone.0298601.s001.zip › Data/地级市Z_F工作报告.zip!/┴╔─■/║∙┬1⁄2╡║╩╨/└┤╫╘╣1⁄2╓┌║┼í╛┬φ┐╦╩2╛▌═°í┐_2016.docx]

各位代表：

　　现在，我代表市人民政府向大会报告工作，请予审议，并请市政协委员和列席会议的同志提出意见。

　　一、“十二五”时期和2015年工作回顾

　　过去的五年，是我市负重前行、奋力开拓的五年。面对严峻复杂的经济形势，在市委的坚强领导下，在市人大、市政协的监督支持下，全市人民万众一心，攻坚克难，较好地完成了各项任务目标。

　　经济发展稳步推进。预计2015年，地区生产总值实现720亿元，年均增长4.2%，三次产业比重为13∶43∶44。一般公共预算收入完成57.1亿元，年均增长0.4%。固定资产投资完成190.4亿元，年均增长-13.8%。社会消费品零售总额实现435亿元，年均增长13.3%。城镇、农村居民人均可支配收入分别为24768元和10233元，年均增长11.4%和12%。

　　改革开放不断深入。政府部门由36个精减到32个;部门预决算和“三公”经费预决算全面公开;完成中冶葫芦岛有色股份制重整;实现土地流转81万亩。实际利用外资、引进内资、出口创汇分别是“十一五”的5.7倍、3.1倍和1.5倍。泳装和船舶基地被评为省级出口基地，独联体海关联盟跨境电子商务综合服务中心落户我市。

　　重点项目加快建设。亿元以上项目563个。徐大堡核电、猴山水库等4个项目列入国家新一轮振兴东北首批重点支持项目。锦西石化120万吨催化汽油加氢深度脱硫装置、绥中电厂二期、方大锦化12万吨离子膜烧碱等370个重点项目建成投产。大润发、沃尔玛等商贸综合体投入运营。

　　基础设施日趋完善。全市公路里程达到9095公里，新增黑色路面2588公里。兴建高速公路建成通车，东戴河动车站、龙湾大街南延拓宽改造、海滨栈道等一批城建项目投入使用。城市外环路工程进展顺利。绥中港区3个5万吨级通用泊位投入使用。葫兴一体化格局初步形成，全市城镇化率达到50%，比“十一五”末提高7个百分点。

　　生态环境持续改善。全市森林覆盖率达到36.2%，城市绿化覆盖率41.9%，荣获国家森林城市称号。建成污水处理厂8座，污水收集率和处理率达到85%以上，重点工业企业污水达标排放率100%。二氧化硫、氮氧化物等4项主要污染物排放完成“十二五”控制指标。虹螺山、白狼山晋升为国家级自然保护区，兴城被评为中国宜居小城，我市成为“最美中国·文化旅游目的地城市”。

　　社会事业协调发展。义务教育省级标准化学校比例达到78%，学前三年毛入园率达到88%，渤海船院新校区建成使用。基层医疗机构全部实施基本药物制度，11项基本公共卫生服务惠及城乡居民。城乡文化建设协调推进，群众体育蓬勃发展。东大杖子战国古墓群入选2011年全国十大考古新发现，新增沙锅屯遗址等7处全国重点文物保护单位。取得省级以上科技成果31项，获得国家发明专利365件，东戴河成为国家火炬计划数字技术特色产业基地。我市成为全国文明城市提名城市，荣获全国双拥模范城“四连冠”。

　　民生质量显著提高。城镇登记失业率连续5年低于省控指标。城乡居民养老保险参保率94.6%，新农合参合率99%以上。连续五年提高城乡低保标准。养老床位突破1万张。完成保障性安居住房7.6万套，改造农村危房1.5万户。38万农村贫困人口实现脱贫。连续8年荣获全国综治工作优秀市称号。

　　各位代表，刚刚过去的2015年是“十二五”收官之年。一年来，我们始终坚持稳中求进工作总基调，狠抓稳增长促投资惠民生各项工作，实现了经济企稳回升、社会和谐稳定。(一)招商引资上项目实现新突破。全年重点项目复工115个，开工45个，竣工40个。青山水库供水工程全部完工，具备向城区供水条件。绥中电厂成功改接华北电网，渤船重工核电装备制造项目试生产。徐大堡核电完成开工准备工作，猴山水库大坝完成总工程量80%，葫芦岛铝业、大唐热电、兴隆大家庭城市综合体等项目陆续开工建设。国家能源科技重大示范工程中船重工海洋核动力平台项目落户我市。

　　抢抓京津冀协同发展重大机遇，健全工作机制，制定承接规划，出台奖励办法，承接京津冀产业、科技和人才转移工作取得初步成效。东戴河·中关村科技产业园成为承接中关村、中科院产业转移和科技成果转化基地。兴城大红门物流中心一期工程投入使用，北京语言大学基础教育中心建设进展顺利，东戴河国家级生物产业基地、鲸兆国际交易广场等8个项目签约落地，北京资源亿家科技园等5个项目签订合作意向，北京中科复兴气垫船等44个项目有序推进。与中关村发展集团及其他城市共同发起设立中关村协同创新母基金。我市成为全省承接北京非首都功能疏解重点城市。积极参与中国绿公司年会、沪企辽宁行、民贸会辽宁行、台湾周等招商活动，引进戴河湾旅游综合体、月星国际建材博览城等项目12个。全年到位内资220亿元。(二)工业经济取得新成效。深入开展“三联系”“双进双解”等活动，成立驻葫央企服务工作组。工业产品产销率保持在98%以上，工业用电量增幅位居全省前列。全年原油加工量增长4.1%;锌锭、烧碱、硫酸产量分别增长1.6%、11.1%、58.5%;发电量增长3.1%。

　　国家级高新技术企业达到32家，高新技术产品产值占规模以上工业总产值15%。实施重点技改项目22个，西门子SST-400型蒸汽轮机实现本地化生产，绥中电厂完成东北地区首台超低排放机组改造，渤船重工荣获省长质量奖。17家企业22项产品被评为省“专精特新”产品。

　　(三)服务业再创新佳绩。省级服务业集聚区和服务业领军企业发展迅速，百大乐园、银象宁远城等项目投入运营。预计服务业增加值实现322亿元，增长5%。

　　电子商务交易额60亿元，增长20%，网商网店突破8000家。中国馨予互联网物联网一体化电商服务平台投入运营，兴城电子商务产业园被评为省级电子商务示范集聚区，兴城、绥中成为全国电子商务进农村示范县。我市被工信部列为“车联网”试点市。全市存款余额1413亿元，增长9.8%;贷款余额856亿元，增长6.3%。

　　东戴河海滨景观工程投入使用，泉海御龙湾等项目开工建设，启动龙湾海滨—觉华岛、绥中长城两个5A级景区创建工作。成功举办泳博会、菩提文化旅游博览会、葫芦文化节。12列动车组分别冠名“葫芦岛号”“东戴河号”。我市成为全国旅游避暑城市，绥中成为中国最美宜居宜业宜游城市，觉华岛荣获中国最美海岛称号。实现旅游总收入147.2亿元，增长10%。

　　(四)现代农业迈出新步伐。产业化龙头企业达到245家，省级示范家庭农场23家，市级以上示范农民合作社245家。绥中成为国家级现代农业示范区。中国农科院兴城果树研究所成为中国北方果树良种苗木繁育和技术转化中心。累计认证无公害农产品145个、绿色食品53个，兴城多宝鱼、寒富苹果、绥中白梨荣获第13届中国国际农交会金奖。

　　完成荒山造林70万亩、矿山生态治理2353亩。实施节水滴灌25万亩。建成饮水安全工程119处，解决8.5万人饮水安全问题。农机总动力168万千瓦，耕播收综合机械化水平达到73%。

　　(五)城乡建设取得新进展。完成龙湾公园、新华立交桥、海日桥改造工程，新老城区排水、燃气、供热设施改造一期工程竣工。完成渤船重工、中冶葫芦岛有色职工生活区供水接收和改造工作。深入开展宜居乡村创建工作，建成市级宜居示范村32个、宜居达标村251个、省级生态镇7个、省级生态村29个。实施农村公路改造469公里。启动龙兴国家湿地公园工程。加强水源地保护区污染源整治，近岸海域水质达标率100%。完成大气污染治理项目258个。建昌被评为省级生态县，列入京津冀生态协同圈。

　　(六)深化改革进入新阶段。市本级行政审批权限由394项削减到188项，各部门权力清单、责任清单向社会公开，聘请100名软环境监督员进行全程监督，开展行政审批服务“大篷车”活动。全面实行“一照一码”“先照后证”，取消缓征各种收费项目83项，新增登记市场主体2.7万户。成立市级不动产登记机构，设立产业(创业)引导基金和招商引资基金，政府和社会资本合作(PPP)顺利推进。

　　(七)社会事业获得新发展。切实推进义务教育均衡发展，改造农村薄弱学校20所，创建省级标准化学校96所，新改扩建公办幼儿园67所。启动县级公立医院改革，培训红十字急救员2.5万人，市妇幼保健院开工建设，市中心医院龙湾院区一期工程投入使用，该工程荣获“鲁班奖”。成功举办第四届全民运动会，乡镇街道文体站、社会体育辅导站和社区书屋实现全覆盖。(八)民生保障再上新水平。全年民生支出133.1亿元，占全市总支出79.3%，同比提高3.1个百分点。机关事业单位基本工资标准、企业退休人员养老金、小学和初中班主任津贴全部上调。城市和农村低保标准分别增长7.2%和10%。大病保险全面铺开，实现新农合与大病保险“一站式”报销。完成棚户区改造4138户、农村危房改造1641户。龙港区郝家屯村棚改实现和谐拆迁。8.4万贫困人口实现脱贫。政府向全市人民承诺的十大民生工程、33件民生实事全部完成。

　　(九)民主法治和政府自身建设迈上新台阶。自觉接受人大法律监督、政协民主监督和社会监督。办理人大代表建议120件、政协提案531件，办复率、见面率100%，满意和基本满意率99.8%，落实和基本落实率69.3%。坚持依法行政，严格执行重大决策法定程序，聘请政府法律顾问，推进政府决策民主化、科学化、法制化。

　　深入开展安全大调研大排查大整治等活动，健全完善规章制度体系和五级安全网格化监管责任体系。加强市场物价网格化监管，强化食品药品日常监管和专项整治，农产品质量安全监管站实现乡镇全覆盖。建成立体化社会治安防控体系，治安环境进一步优化。

　　落实重大事项定期报告制度，建立工作对接沟通机制，实施行政领导干部问责制，对不作为不担当、庸政懒政怠政以及各类安全事故等相关责任人进行了严肃追责。加快推进社会信用体系建设，制定失信企业惩戒联动实施办法。加大信访工作力度，切实维护人民群众合法权益，认真解决拖欠农民工工资等热点难点问题。整合信息平台资源，提高12345政府热线服务水平。强化审计监管和预算约束，规范政府收支，全市“三公”经费同比下降16.7%。扎实开展“三严三实”专题教育，政府工作作风进一步好转。

　　民族宗教、对外交往、国家安全、新闻出版、妇女儿童、国防动员、边海防、民兵预备役、气象服务、慈善救助、应急管理、统计、人防、地震、供销、住房公积金、侨务、档案、地方志等工作成绩显著。

　　各位代表，这些成绩的取得，是省委、省政府和市委坚强领导的结果，是市人大及其常委会依法监督、市政协民主监督和社会各界监督支持的结果，是全市人民齐心协力、艰苦奋斗的结果，是各级干部尽职尽责、扎实工作的结果。在此，我代表市政府，向全市各族人民，向人大代表、政协委员和老领导、老同志，向各民主党派、工商联、无党派人士、人民团体和社会各界人士，向中省直单位、驻葫部队指战员、武警官兵、公安民警，向在座的各位同志，向在葫芦岛投资创新创业的广大客商，向所有关心支持葫芦岛发展的海内外朋友，表示最衷心的感谢和最崇高的敬意!

　　在总结成绩的同时，我们也清醒地认识到我市当前面临的形势和工作中存在的问题。世界经济复苏低迷，经济下行压力依然很大，摆在我们面前的困难和挑战前所未有。我市经济发展质量和效益不高，经济增长乏力，发展方式粗放，经济结构不合理;生态环境压力加大，节能减排任务繁重;安全生产形势严峻，安全事故时有发生;财政收支矛盾突出，政府性债务进入偿还高峰期;一些干部思想观念守旧，开放意识不强，适应新常态、引领新常态的能力不足;一些干部缺少责任意识和担当精神，工作作风仍需进一步转变;依法行政、简政放权做得还不到位，经济发展软环境亟待改善。对这些问题，市政府将采取切实有效的措施，认真加以解决。

　　各位代表，党的十八届五中全会提出创新、协调、绿色、开放、共享的发展理念，是针对我国经济发展新常态、世界经济复苏低迷开出的药方。新的发展理念就是指挥棒，我们要以五大发展理念为总指南、总抓手，加大供给侧结构性改革力度，全面落实各项改革发展任务，坚持以经济建设为中心，坚持以提高发展质量和效益为中心，坚持以人民为中心，切实解决经济社会发展中的深层次问题。我们也深刻认识到，我国经济发展长期向好的基本面没有变，经济韧性好、潜力足、回旋余地大的基本特征没有变，持续增长的良好支撑基础和条件没有变，经济结构调整优化的前进态势没有变。新一轮振兴东北、京津冀协同发展、环渤海地区合作发展、一带一路建设等国家战略的深入实施，为我市振兴发展提供了重大历史机遇。多年来，在历届领导班子和全市人民的共同努力下，我市具备了较为坚实的发展基础，积累了许多宝贵经验。只要我们坚定地干、大胆地干、扎实地干、精准地干，就一定能实现葫芦岛又好又快的发展!

　　二、“十三五”时期主要目标任务和2016年工作安排

　　今后五年，是我市全面建成小康社会的决胜期。政府工作总体思路是：坚持以党的十八大和十八届三中、四中、五中全会精神为指导，深入贯彻落实习近平总书记系列重要讲话精神，牢固树立创新、协调、绿色、开放、共享五大发展理念，以“五位一体”总体布局和“四个全面”战略布局为统领，以“四个着力”为主线，坚持稳中求进工作总基调，大力实施改革引领、创新驱动、结构优化、文化兴市、生态立市五大发展战略，深化供给侧结构性改革，提高发展质量和效益，保持战略定力，增强发展自信，坚决打胜脱贫攻坚战，建设生态宜居美丽富庶滨城，到2020年全面建成小康社会。

　　今后五年的预期目标是：地区生产总值年均增长7%;一般公共预算收入、固定资产投资、社会消费品零售总额分别年均增长5%、10%、10%;城镇、农村居民人均可支配收入分别年均增长8%。

　　今后五年的主要任务是：

　　全面建设富庶葫芦岛。推进农村一二三产业融合发展，推动传统产业向中高端迈进，增强战略性新兴产业和创新服务业支撑作用，打造文化旅游支柱产业。加快县城、新市镇和美丽乡村建设，加速构建葫兴同城化大城区格局，实现新老城区共建共享共同发展，到2020年全面完成现有棚户区、城中村和农村危房改造，龙港区率先实现全域城镇化，全市城镇化率达到60%以上。坚决打胜脱贫攻坚战，确保建昌县“摘帽”，确保全市16万贫困人口稳定脱贫。全面提高公共服务共建能力和共享水平，满足多样化民生需求，让改革发展成果更多更公平惠及广大人民群众，使人民群众在共建共享发展中有更多获得感。

　　全面建设创新葫芦岛。深化重点领域改革，破除影响市场经济发展的各种阻碍。深入实施“互联网+葫芦岛”行动计划和“中国制造2025”葫芦岛实施方案，强化企业创新主体地位，加强知识产权保护利用，促进新产业、新业态、新模式蓬勃发展。加快创新创业平台建设，加强人才培养引进，健全扶持激励政策，鼓励大众创业万众创新，让每个人都有创新创业的空间，让每个人都有出彩的机会。

　　全面建设开放葫芦岛。坚定不移实施招商引资、招才引智、招科引技，加快融入京津冀协同发展、一带一路和环渤海地区合作发展，把东戴河建设成为承接京津冀产业、科技、人才转移的先导区和示范区，确保承接京津冀产业转移工作取得实质性进展。积极参与国际产能和装备制造合作，壮大外向型产业集群，加快海外仓储物流中心建设，大力发展港口经济、临港经济等海洋经济，建设海洋经济强市。外贸出口、引进内资实现翻一番。

　　全面建设文明葫芦岛。坚持用中国梦和社会主义核心价值观凝聚共识。加强思想道德建设和社会诚信体系建设，弘扬爱国主义、集体主义、中华优秀传统文化和中华传统美德，营造向上向善、诚信互助的社会风尚。开展全国文明城市、全国卫生城市和国家生态市“三城联创”活动，为葫芦岛振兴发展汇聚强大精神动力。

　　全面建设美丽葫芦岛。坚持绿色发展理念，加快建设资源节约型、环境友好型社会，促进人与自然和谐发展。加强生态文明建设，深入实施青山、碧水、蓝天、沃土工程，实行最严格的环境保护制度，筑牢生态安全屏障。到2020年，城市空气质量优良天数比例达到80%以上，森林覆盖率达到37.5%，城市绿化覆盖率达到44.3%，垃圾无害化处理率达到100%。

　　各位代表，建设生态宜居美丽富庶滨城，是全市280万人民的共同愿景。实现这一共同愿景，我们必须进一步解放思想，以开拓创新的精神加快发展;必须抢抓机遇，以昂扬向上的斗志奋勇争先;必须敢于担当，以脚踏实地的作风履职尽责;必须苦干实干，以坚韧不拔的意志攻坚克难。我们坚信，只要坚持，梦想总是可以实现的，葫芦岛的明天会更加美好!

　　各位代表，2016年主要任务目标是：地区生产总值增长5%;一般公共预算收入增长2.1%;固定资产投资增长10%;社会消费品零售总额增长8%;城镇、农村居民人均可支配收入分别增长7%和8%。

　　实现上述目标，我们要努力做好以下十个方面工作。

　　(一)全力推进项目建设年。发挥有效投资对稳增长调结构的关键作用，把项目建设作为葫芦岛经济发展的生命线，一切围绕项目干，实现“十三五”攻坚发展的开门红。全年计划实施亿元以上项目182个，总投资2020亿元。其中：工业项目36个，总投资304亿元;服务业项目112个，总投资1073亿元;农业项目4个，总投资17亿元;基础设施建设项目30个，总投资626亿元。

　　建立完善项目建设工作机制。建立重大项目推进工作机制，完善领导干部包扶重点项目制度，强化组织领导，层层落实责任。建立考核激励机制，以项目建设实绩评先选优。建立约谈问责机制，督促工作、鞭策后进。建立项目储备机制，实施项目库动态管理。建立项目拉练机制，加强督查调度，确保项目建设年取得实实在在的成效。

　　切实加强经济发展要素保障。强化银企对接合作，积极培育企业通过新三板上市等渠道直接融资，发挥产业引导基金作用，争取上级资金支持。积极探索农村集体建设用地入市等新模式，继续开展增减挂钩试点，有效盘活存量土地，满足项目建设的资金、土地等要素需求。(二)加速工业转型升级。继续深入开展“三联系”“双进双解”活动，落实市领导帮扶重点企业责任制，促进企业降成本、去产能，努力做优存量、做大增量。

　　提升装备制造产业实力，加快推进中船重工海洋核动力平台、葫芦岛铝业、渤船海工装备基地、首钢东华厂高铁桥梁支座等重点项目建设。优化能源电力产业结构，加快大唐热电一期工程建设进度，力争开工建设徐大堡核电一期、大唐新能源高桥风力发电等重点项目，积极推进中港建设集团燃气发电供热、兴城抽水蓄能电站、南票煤电热电联产升级改造等项目尽快实施。推动石油化工产业升级，加快锦西石化汽油和柴油国Ⅴ标准质量升级、南票膨润土(紫砂)精细加工产业园、盛坤碳纤维复合材料等重点项目建设。加快有色冶炼产业扩能改造，加速15万吨电解铜和有色金属综合回收等重点项目建设。

　　大力支持民营经济发展。没有葫芦岛民营经济的快速发展，就没有葫芦岛的全面振兴。我们要认真落实国家和省市各项扶持政策，加快推进民营经济做大做强。我们要为民营经济搭建融资平台，建立政府、银行、企业三方联席会议制度，积极开展银企对接活动，帮助民营企业解决融资难题。我们要为民营经济搭建协作平台，引导民营企业与驻葫央企开展协作配套，拓展延伸上下游产业链。我们要为民营经济搭建科研平台，发挥驻葫高校和央企科技人才优势，建立完善产学研用合作机制，推动科技成果转化。我们要为民营经济搭建信息服务平台，完善信息服务功能。我们要为民营经济搭建对接平台，充分发挥工商联和各类商会协会作用，建立政府与民营经济对接沟通机制，为民营经济排忧解难。我们要建立扶持民营企业发展激励机制，树立一批社会贡献大、企业信誉好的民营企业先进典型。(三)推动服务业创新发展。以文化旅游产业为支撑，大力发展现代服务业，加快构建以服务业为主导的经济发展新格局。

　　推进传统服务业提档升级。加快服务业集聚区建设，积极发展城市“夜经济”，促进商贸物流产业繁荣发展。加快首开滨海、隆东国际两个城市综合体项目建设，力争兴隆大家庭项目主体完工，万达广场项目开工建设，连山五金机电城等项目投入运营。依托金岸龙湾等服务业集聚区，推动泉海御龙湾、首山丽汤等特色地产项目建设，走房地产业与休闲旅游、健康养生等产业有机衔接、良性互动的发展新路子。

　　加速服务业新兴业态发展。拓展电子商务应用领域，扩大在线交易规模，年内网商网店突破1万家、电商交易额突破80亿元。推广运用物联网、大数据、云计算等新一代信息技术，全球化工新材料大数据分析系统稳定运营，百搭智能穿戴生态系统投入使用。扶持金融产业发展，规范金融市场秩序，防范金融风险，提高金融服务实体经济水平。加快文化创意产业发展，繁荣会展经济，打造泳博会、泵博会、菩提文化旅游博览会、葫芦文化节等知名会展品牌。

　　做强文化旅游支柱产业。树立大旅游理念，依托城泉山海岛河等丰富旅游资源，强化全市旅游资源整体规划开发，实现由滨海游向全域游转变;补齐旅游项目短板，加快富都红麦坊主题乐园、宏跃旅游大世界等四季旅游项目建设，实现由季节游向全年游转变;丰富旅游产品和文化内涵，加快建设葫芦山庄文化创意产业园、滨海露营公园等文化旅游项目，建昌白狼山、画廊谷、龙潭大峡谷等生态旅游项目，实现由传统观光游向休闲度假、健康养生游转变。加快古城、长城、碣石遗址等名胜古迹保护展示利用，打造历史文化旅游名城;加快推进戴河湾旅游综合体、银泰水星海洋乐园、海滨栈道三期工程等特色旅游项目建设，打造滨海旅游精品;加强塔山革命烈士陵园、辽西第一党支部旧址等爱国主义教育基地建设，打造红色旅游品牌。加快龙湾海滨—觉华岛、绥中长城两个5A级景区创建工作。推进文化旅游体制改革，完善旅游交通规划，加强旅游基础设施建设，规范旅游市场管理，提升旅游服务质量和水平，建设全国旅游休闲养生度假目的地城市。(四)加快发展现代农业。坚持重农固本，加大强农惠农富农力度，推进农业供给侧结构性改革，着力构建农业与二三产业交叉融合的现代产业体系，实现农业增效、农民增收、农村繁荣。

　　调整优化农业结构。完成土地确权120万亩，新增土地流转面积60万亩。重点抓好兴城省级现代农业综合示范区、建昌万亩桃园等6个现代农业主导产业示范区，建设设施农业标准园10个，调减玉米种植面积，新增优质粮油作物11.5万亩。建设标准化生态健康养殖场100个。依托中国农科院兴城果树研究所，以前所现代生态农业示范区、大台山果树农场为龙头，大力发展特色林果经济，建设东北果业强市。

　　提升农业产业化水平。培育和引进新型农业经营主体，新增省级农民示范合作社15个、市级农民示范合作社30个、家庭农场100个，培育市级农业科技创新团队10个。开展职业技能和实用技术培训，培养新型职业农民。大力推进“互联网+农业”，加快“农村淘宝网”等电子平台建设，发展智慧农业，注重农业生产全产业链条管理，推广观光农业、休闲渔业、体验式农场等农业营销新业态。积极推进农产品出口，发展创汇农业。大力实施品牌战略，提高绿色有机和地理标志商标产品的质量和产量，构建数量充足、品种和质量契合消费者需求的农产品供给体系。制定奖励政策，鼓励企业加强品牌包装和市场营销，建设面向京津冀和东北地区特色农产品供应基地。

　　改善农业生产条件。加强农田水利基本建设，继续实施小农水工程。完成节水滴灌15万亩、高标准农田综合整治1.3万亩。提高农业机械化水平，耕播收综合机械化率达到74%。加强防雹减灾工作，实现人工增雨常态化。

　　(五)提升开放创新水平。坚持把招商引资上项目作为长期性战略任务，抢抓重大政策机遇，丰富对外开放内涵，完善创新体制机制，以开放创新推动发展动力转换。

　　全面扩大开放。主动融入京津冀协同发展、环渤海地区合作发展，强化以秦皇岛市为重点的区域经济合作，健全完善招商引资激励和保障机制，加强队伍建设和人才培养，重点围绕长三角、珠三角、东三省、港台、日韩等国家和地区，实施精准招商、实体招商。把驻葫商会作为政府招商分支机构，强化以商招商。整合开发区(园区)资源，理顺开发区体制，完善开发区功能，明确发展定位，依托工业园区，实施产业招商。加快推进44个京津冀产业转移在谈项目和沪企、民企、民贸会辽宁行签约项目落地。面向一带一路沿线国家和地区开展定向招商、进出口贸易、产能合作和海外并购，积极争取西门子等外企增资扩产。今年引进内资、利用外商直接投资分别增长10%以上，外贸出口增长5%左右。

　　推动创新创业。整合全市科研机构和重点企业科研资源，强化与国家大学科技园长期深度合作，探索政产学研用合作新模式，促进科技成果转化。建设东戴河高科技人才休闲度假区，吸引高科技人才“东戴河度假”。大力引进国内外高端人才，全年引进域外研发团队5个，新增高技能人才1000人。加快建设青牛软件产业园、京东高新技术产业园、天马辉光电产业园等项目，中关村科技成果转化基地开工建设。整合政府信息服务资源，建立市大数据中心，运用大数据技术推动创新创业，提升政府治理能力。实施大众创业万众创新，依托辽工大、辽宁财贸学院、渤海船院和中国农科院兴城果树研究所为葫芦岛培养创新创业型人才，加大政策扶持力度，发挥资金引导作用，鼓励在葫大学毕业生就地创新创业，鼓励科研团队和研发机构利用高新区国家级科技孵化器和辽宁财贸学院创新创业园等载体和平台，开展众创空间试点，加快推动“双创”基地建设。

　　(六)推进城乡协调发展。加快新型城镇化进程，加速葫兴同城化，不断提升城乡环境质量、人民生活质量和城市综合竞争力。

　　持续改善生态环境。加强海岸线保护和治理，巩固拓展“创森”成果。开工建设龙兴国家湿地公园，加快推进水环境综合整治及生态修复项目。开展尾矿库专项整治，消除安全隐患，修复生态环境。开展治气治霾专项治理，重点整治企业大气污染、扬尘污染和移动源污染，推进燃煤锅炉升级改造，淘汰城区10吨以下老式锅炉，加快淘汰黄标车，推广新能源汽车，实现新老城区公交“绿色化”。大力发展绿色低碳循环产业，启动国家级环保产业园项目。支持重点企业实施节能环保技术改造，着力推进锦西石化氮氧化物减排、废氢节能利用等项目。加强环境治理，严格执行环保法律法规，清理整顿环保违规建设项目。鼓励公众参与环保事业，形成政府企业社会共治的环境治理体系，设立环境治理举报奖励基金100万元。重点企业污水达标排放率100%，全年城市空气质量优良天数占比达到75%以上。

　　加快城乡基础设施建设。启动滨海公路升级改造和葫芦岛港疏港铁路项目，开工建设绥中港区2个5万吨级通用泊位。开工建设辽西北供水二期葫芦岛配套工程和青山水库环保工程，完成猴山水库年度建设任务，实现龙屯水库向绥中城区供水。加快建设城市外环路，开工建设龙湾海滨—觉华岛陆岛码头，实施葫芦岛北站改扩建工程。开展城市地下管网普查，规划建设城市地下综合管廊。

　　加快推进城镇化进程。探索农业转移人口市民化路径，健全土地、财政、教育、就业、医疗、养老、住房保障等配套措施，促进有能力在城镇稳定就业和生活的常住人口有序实现市民化。以满足新市民住房需求为出发点，把化解房地产库存与推进城镇化有机结合，逐步消化商品房库存，改善居民住房条件。

　　提高城乡治理水平。完成城市总体规划修编和各类专项规划，开工建设城市规划综合展览馆。开展“爱海洋、爱蓝天、爱青山、爱家园”主题活动，大力实施绿化美化亮化工程，把好山好水好风光融入城市。在驻葫部队倡导开展“热爱第二故乡、建设第二故乡”活动，鼓励和号召部队官兵积极投身地方建设。进一步推动“宜居乡村”建设，实施村内道路建设项目359个、美丽乡村建设项目175个，建设少数民族特色村镇3个。强化城市精细化管理，建设数字化城市管理中心和综合性城市管理数据库，建设智慧城市。理顺城市管理体制机制，提升供水供暖、园林环卫等市政服务水平。创新物业管理体制机制，提高物业管理质量水平，增强居民缴费意识，完善社区服务功能，建设“10分钟社区服务圈”。

　　(七)着力保障改善民生。完善社保体系。新增实名制就业8000人，农村劳动力转移就业30万人，零就业家庭保持动态为零，城镇登记失业率控制在4%以内。低保标准平均提高10%，实现城乡低保取暖救助全覆盖。巩固新农合成果，提高大病保险报销比例。完成机关事业单位养老保险改革。加大社会保险扩面征缴力度，加快推进医疗保险城乡统筹。

　　优化公共服务。强化学前教育公益性和普惠性，推进义务教育均衡发展、高中教育内涵发展。完善中高等职业教育体系，加强新市民职业技能培训，建设家政服务培训基地，打造葫芦岛“家政名嫂”品牌。推进健康葫芦岛建设，加快县级公立医院综合改革，健全社区(村屯)卫生服务网络，建设龙港正业健康产业示范园区，完成市中心医院龙湾院区二期工程。提高教育、医疗等公共服务体系承载能力，积极应对全面“二孩”政策带来的人口增长。开展全民健身活动，繁荣文化事业，完善乡村社区文化体育设施。加强文物保护利用，深入挖掘233处县级以上文物保护单位、1529处不可移动文物历史内涵，讲好葫芦岛故事。

　　保障社会和谐稳定。坚持安全发展理念，培养全民安全文化意识，设立安全隐患举报奖励基金100万元。建立安全隐患信息平台和重大安全隐患预警系统，健全完善安全生产专家组等规章制度，加强安监机构队伍建设，落实安全生产主体责任，强化安全隐患整改和督查问责，坚决遏制重大生产安全事故发生。加强校园安全工作，提高师生安全意识和防范能力。保障食品安全，全面启动食品安全放心县(市)区建设。落实领导干部信访接待日和包案制度，加强信访法律援助，维护群众合法权益。创新立体化社会治安防控体系，加强军警、军民联防联控，提升应急处置能力，坚决打击“黄赌毒”，建设平安葫芦岛。

　　各位代表，2016年，市政府将集中力量做好十大民生工程，办好30件民生实事。

　　一是城区饮水工程。建设青山水源净水厂，完成城区供水管网改扩建二期工程，建设市水质检测中心。二是市政提升工程。实施龙背山生态公园改造一期工程，完成葫芦岛站广场改造，完善城区小街小巷市政设施。三是道路畅通工程。开工建设新老城区快速干道(泰康路改造)，实施龙绣街、化机路和葫六线城区段改造工程。四是环境治理工程。实施茨山河上游综合治理和五里河上游生态修复一期工程，完成中冶葫芦岛有色烟气脱硫治理、锦西石化污水车间恶臭气体治理等工程。五是老旧小区改造工程。加强物业弃管小区综合整治，实现弃管小区电梯安全维护保险全覆盖，完成 100万平方米“暖房子”改造工程。六是保障性安居工程。改造城市棚户区4188户、农村危房1245户，新建公租房600套。七是惠民便民工程。开展农超对接，建设配送中心和连锁超市。完成900户残疾人家庭无障碍改造，新建“妇女儿童之家”10所。建设城区便民公厕24座。八是教育助学工程。妥善安排农民工子女入学，在新区和老区各建一所设施完备、功能齐全的综合性幼儿园。九是公共卫生工程。建设市结核病防治所综合楼和200个标准化村卫生室。十是养老设施工程。维修改造农村敬老院12所，新建社区老年人日间照料中心和农村幸福院58所。(八)坚决打胜脱贫攻坚战。认真贯彻落实中央、省市扶贫工作会议精神，举全市之力帮扶以建昌为重点的贫困地区稳定脱贫。全年落实种植、养殖和加工类规模经营项目120个，开展扶贫培训5000人次，加大贫困人口医疗救助力度。大力开展智力、社会、交通、旅游扶贫，每年定向培养脱贫致富带头人150人，创新金融扶贫措施，加强驻村定点帮扶工作，加快贫困地区三级路网建设，发挥旅游景区开发扶贫带动作用，确保4.7万建档立卡贫困人口脱贫。(九)优化经济发展软环境。市政府将下定决心、下真功夫、下大力气，创造优良的政务环境、投资环境和法治环境，不断提升葫芦岛振兴发展的核心竞争力。建立软环境建设行政“一把手”负责制和问责制，严格执行政府部门权力清单、责任清单、负面清单。加大简政放权力度，建设市行政服务中心，全面推进并联审批，建立项目联合审批办公日制度。完善“保姆式、兜底式”服务机制，设立企业服务代办中心和项目落地代理中心，实现政府职能由管理型向服务型转变。制定政府系统首办(首问)责任制实施办法，提高政府机关工作质量和效率。理顺部门职能，整顿机关作风，加强督查检查，严厉查处破坏软环境的行为。建立软环境公众微信平台，提高公众参与度，形成“人人都是软环境”的发展氛围。加强社会诚信体系建设，建设诚信葫芦岛。(十)加强民主法治和政府自身建设。自觉接受人大及其常委会的监督，严格执行各项决定决议，定期向人大及其常委会报告工作，积极支持人民政协履行政治协商、民主监督、参政议政职能，切实做好人大代表建议和政协提案办理工作。认真听取各民主党派、工商联、无党派人士和各人民团体的意见和建议。

　　依法全面履行政府职能，严格落实重大决策法定程序。健全完善海洋保护、旅游市场、城市规划等领域地方政府规章和规范性文件体系，完善行政案件责任人出庭应诉制度。整合绩效考核、目标管理和干部考核制度，建立科学高效、考核结果运用合理的考评机制，提高政府执行力。深入开展“葫芦岛讲坛”活动，建立人才引进培养交流机制，提高公务员队伍整体素质。

　　加大国资国企改革力度，加快机关事业单位改革。深化投融资体制改革，防范和化解政府性债务风险。提高公共财政管理水平，牢固树立过紧日子的思想，着力增收节支，增强民生保障能力。强化政务公开，推行阳光政务。加强对县(市)区管理，大力扶持县域经济发展。

　　切实加强政府自身建设，坚持法定职责必须为、法无授权不可为，把法治思维和法治方式贯穿到政府工作的全过程。深入践行“三严三实”，严格执行中央八项规定，坚决反对“四风”，从严整治“不作为不担当”“庸政懒政怠政”行为。贯彻落实党风廉政责任制，推进政府系统惩治和预防腐败体系建设。减少会议数量，提高会议质量，严把发文关口，力戒文山会海。强化督查督办和约谈问责，狠抓政府各项工作落实，坚决做到言必信、行必果，切实提高政府公信力和执行力，努力建设法治政府、诚信政府、服务政府、责任政府、廉洁政府。

　　各位代表，“十三五”的宏伟蓝图已经绘就，振兴发展的冲锋号角已经吹响。让我们在省委、省政府和市委的坚强领导下，紧紧依靠全市各族人民，励精图治、开拓创新，用激情、智慧和汗水创造无愧于历史、无愧于人民、无愧于时代的崭新业绩，为建成生态宜居美丽富庶滨城、夺取全面建成小康社会的伟大胜利而努力奋斗!
